# Pictorial Review of Paediatric Limp

**DOI:** 10.3390/pediatric17010014

**Published:** 2025-01-27

**Authors:** Shashank Chapala, Sahana Giliyaru, Rajesh Botchu, Suvinay Saxena, Karthikeyan P. Iyengar, Muthusamy Chandramohan

**Affiliations:** 1 Department of Radiology, AIG Hospitals, Hyderabad 500032, India; chapalashashank@gmail.com; 2 Department of Pediatrics, Rajarajeswari Medical College and Hospital, Dr MGR Educational and Research Institute, Kambipura 560074, India; sahanamanjunath@gmail.com; 3 Department of Musculoskeletal Radiology, Royal Orthopaedic Hospital, Birmingham B31 2AP, UK; 4 Department of Radiology, Chirayu Medical College and Hospital, Bhopal 462030, India; suvinaysaxena@gmail.com; 5 Department of Orthopaedics, Mersey and West Lancashire Teaching Hospitals NHS Trust, Southport and Ormskirk Hospital, Southport PR8 6PN, UK; kartikp31@hotmail.com; 6 Department of Radiology, Bradford Teaching Hospitals, Bradford BD9 6RJ, UK; m.chandramohan@bthft.nhs.uk

**Keywords:** pain, paediatric limp, ultrasonography, MRI, radiography, paediatric hip, paediatric gait

## Abstract

A limp is an abnormal, uneven or laboured gait typically resulting from pain, weakness, or structural deformity involving the hip, lower limb, spine or abdominopelvic abnormalities. Limps in children are common and have diverse causes that can be benign to life-threatening including trauma, congenital malformations, and neoplastic diseases. Diagnosis involves identifying gait abnormality thoroughly examining history and physical exam, assessing tenderness and range of motion, and completing targeted lab and radiographic studies. We present an imaging review of various usual and unusual causes of limp in different age groups such as in toddlers (1–3 years), children (4–10 years), and adolescents (11–16 years) with a comprehensive literature review.

## 1. Introduction

Acute limp is a common presentation in the paediatric population in primary and secondary care. Many departments have protocols in place for examination and initial investigation that consider common causes. In conjunction with clinical and laboratory findings, imaging in the form of radiographs and ultrasound are often utilised first-line as the modalities of choice for investigating this group of patients. Common differentials, e.g., transient synovitis is important but occasionally it is necessary to consider other causes, especially with unusual presentations or where symptoms persist despite normal initial imaging investigations. Age-wise common aetiologies of limp in children are listed in Table 1 and various aetiologies of limp in children mentioned by the AAFP are listed in Table 2.

Outline:

We present a case-based pictorial review of some of the musculoskeletal imaging findings of rarer diagnoses that were encountered in an acute teaching hospital in children and adolescents whose main presenting issue was limp.

These include the following:

1. Osteomyelitis of the ischiopubic synchondrosis;
2. Osteochondrosis of the ischiopubic synchondrosis;
3. Haemophagocytic histiocytosis;
4. Juvenile idiopathic arthritis (JIA) of hip;
5. Rectus femoris avulsion fracture;
6. Crystal arthritis of the hip;
7. Osteomyelitis of the iliac crest;
8. Ewing sarcoma of femur;
9. Langerhans cell histiocytosis (LCH);
10. Leukaemia;
11. Simple bone cyst neck of femur with fracture;
12. Septic arthritis of left sacroiliac joint;
13. Discitis of lumbar spine;
14. TB pyomyositis of the thigh;
15. Stress fracture of the sacral ala;
16. Testicular torsion.

Imaging features of a few conditions are included in (Table 2).

## 2. Osteomyelitis of Ischiopubic Synchondrosis

Osteomyelitis of the ischiopubic synchondrosis (IPS) is not uncommon. The bone adjacent to IPS shares characteristics with the metaphysis of short bones. About 30% of haematogenous osteomyelitis cases occur at these metaphysis-equivalent sites in a study [2]. Staphylococcus aureus is the primary cause, but other pathogens like beta-haemolytic streptococci are reported. Diagnosing IPS osteomyelitis through X-rays is challenging due to irregular ossification of normal bone in children, particularly between ages 5 and 8 being the commonest age of occurrence [3]. The IPS typically fuses between 9 and 11 years. Accurate diagnosis requires careful evaluation of radiographic findings, clinical presentation, and the results from other imaging modalities.

**Figure 1:**
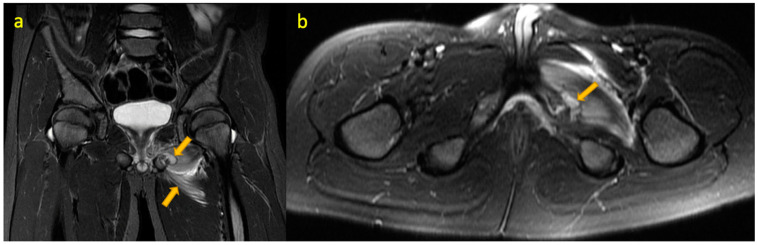
(**a**,**b**) Coronal STIR and axial T2W FS MR images of an 8-year-old boy with osteomyelitis of the left-sided ischiopubic synchondrosis. Note associated soft tissue abscess and muscle oedema.

In cases of suspected osteomyelitis near the IPS, advanced imaging is crucial. CT demonstrates the extent of bone destruction, rarefaction, sequestrum, or involucrum, small sequestra detection is better appreciated on CT than MR, and soft tissue involvement and oedema can be evaluated. MRI (82–100% sensitive in early detection) shows reduced signal on T1W and high signal on T2W and STIR sequences. Other findings include cortical erosions, periosteal new bone formation, and soft tissue involvement (Figure 1). Contrast imaging adds sensitivity, and an abscess will show peripheral enhancement. Bone scintigraphy is rarely used as an adjunct. Imaging reveals increased uptake in perfusion, blood pool, and delayed phases, while uptake more than or equal to triradiate cartilage indicates abnormality [3]. In suspected osteomyelitis, an aggressive diagnostic approach by MRI as the next imaging investigation following radiography is necessary as prompt imaging prevents diagnostic delays and ensures accurate detection of osteomyelitis.

## 3. Osteochondrosis Ischiopubic Synchondrosis

**Figure 2:**
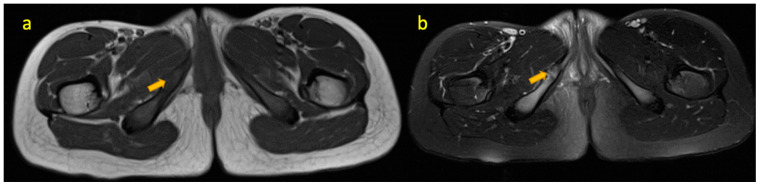
(**a**,**b**) Axial T1W and axial T2W FS MR images of an 11-year-old girl showing mild bone marrow oedema signal around mildly enlarged right ischiopubic synchondrosis consistent with osteochondrosis. There is no surrounding soft tissue collection.

The IPS is a cartilage tissue connecting the ischial and pubic rami ossification centres, which ossifies during puberty. Before ossification, uneven mechanical stress can cause asymmetric expansion, leading to delayed widening and ossification [4]. Some children develop groin and gluteal pain and a limp. This condition, known as “osteochondrosis ischiopubica” (van Neck–Odelberg disease), occurs more commonly between 4 and 16 years of age [5] and is a benign and self-limiting condition (Figure 2). Treatment includes anti-inflammatory drugs, bed rest, and avoidance of exercise.

## 4. Haemophagocytic Lymphohistiocytosis

**Figure 3:**
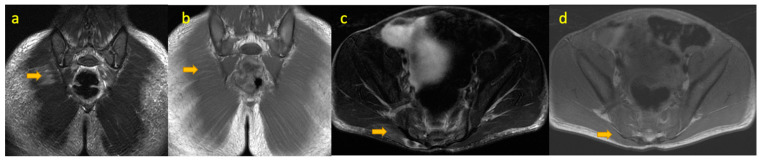
(**a**–**d**)—Haemophagocytic lymphohistiocytosis in an 8-year-old boy who initially presented with 2 weeks’ history of painful right-sided limp and raised CRP. MRI of the pelvis (coronal T1W, STIR, axial T1W, and T2WFS images) reveals focal nonspecific muscle oedema signal and mild enlargement of the right gluteus maximus muscle.

Haemophagocytic lymphohistiocytosis (HLH) is characterised by hyperactivation of the immune system where uncontrolled macrophage activity leads to haemophagocytosis of host cells (RBCs, platelets, WBCs). There is a concurrent impairment of natural killer (NK) cells and cytotoxic T lymphocyte activity to regulate excessive macrophage activity. HLH is classified into two groups: Primary HLH (genetic/familial) is an autosomal recessive disorder that most commonly affects infants and children. It is caused by defects in genes involved in cytotoxic granule exocytosis [6]. Secondary HLH is associated with infections (e.g., EBV, CMV, varicella, measles), malignancies, and rheumatologic conditions with no familial mutations [7]. Secondary HLH can occur at any age [8] (Figure 3).

## 5. Juvenile Idiopathic Arthritis of the Hip

This the most common chronic arthritis in childhood. Symptoms must start under 16 years of age. Oligoarticular or polyarticular arthritis greater than 6 weeks’ duration must be present to diagnose. There are several subtypes: oligoarticular, polyarticular, and systemic-onset (Still disease). A proportion of patients have raised serum rheumatoid factor.

**Figure 4:**
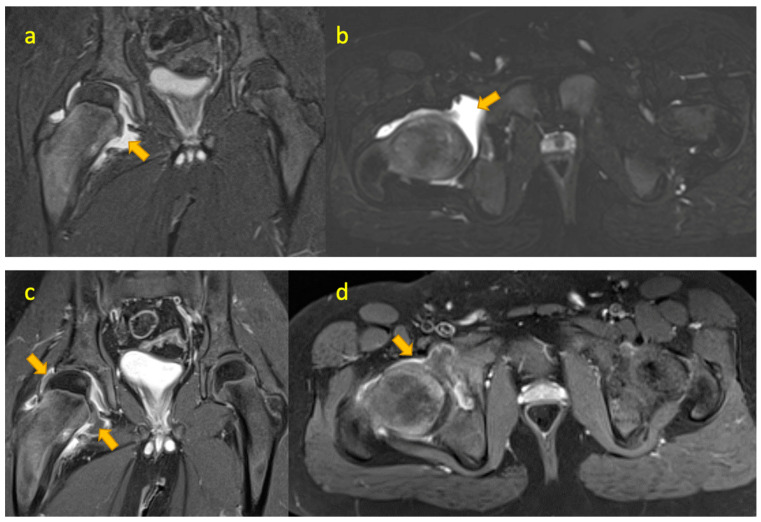
(**a**–**d**) MRI hip of a ten-year-old boy who presented with a painful right-sided limp. Coronal STIR, axial T2W FS, and post-gadolinium fat-saturated coronal and axial MRI images showing a large effusion and diffuse synovitis in the right hip proved to be juvenile inflammatory arthritis.

MRI effectively distinguishes hip arthritis in juvenile idiopathic arthritis (JIA) from arthralgia of unknown origin, confirming JIA diagnosis with high specificity (100%) [9]. Although sensitivity is limited, MRI outperforms clinical diagnosis as reported by El-Azeem et al. [10] and Nistala et al. [11]. Currently, JIA diagnosis relies primarily on clinical findings and laboratory data, with imaging playing a secondary role. However, the Pediatric Rheumatology International Trials Organization (PRINTO) recommends imaging for sacroiliitis evaluation [12]. Frequent MR findings in JIA include synovitis, bone marrow oedema, enthesitis, bursitis, myositis, bone erosion, cyst formation in bone, and chondromalacia [9] (Figure 4). For challenging hip joint clinical assessment, MRI is valuable when radiographs and ultrasound findings are inconclusive and for narrowing down differentials or confirming the diagnosis [13].

## 6. Rectus Femoris Avulsion Fracture

**Figure 5:**
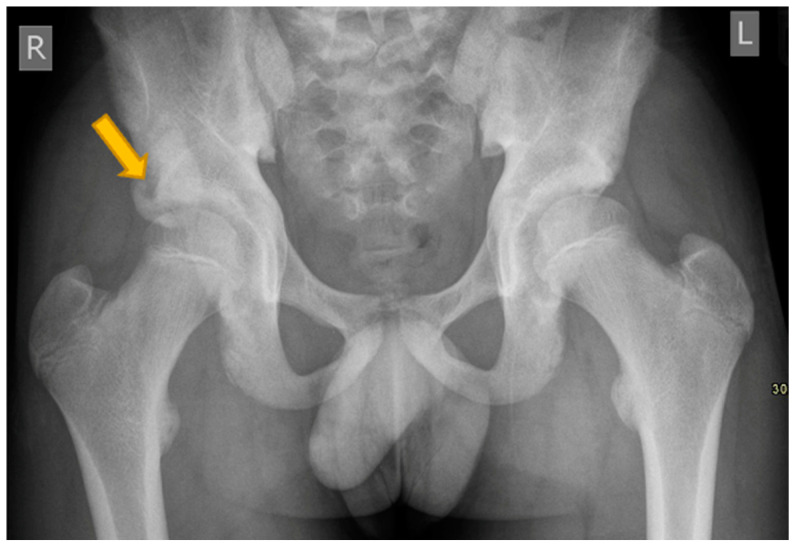
Painful right-sided limp in a 14-year-old male, a keen footballer. A plain radiograph shows an acute avulsion fracture of the right anterior inferior iliac spine.

Initial imaging for pelvic or hip injuries is plain radiographs, which can reveal avulsion fractures at the anterior inferior iliac spine. For rectus femoris injuries, T2-weighted MRI shows proximally retracted avulsed muscle fibres, fluid-filled perifascial/fascial compartments [14], interstitial haemorrhage, and oedema giving a feathery muscle appearance [15]. Kassarjian et al. described dissociation between deep bipennate and superficial unipennate components, resembling a “finger withdrawing from a glove [16]. Hughes et al. described focal oedema and fluid at the myotendinous junction as a “bull’s-eye lesion” [17]. Overall advantages of MR imaging include accurate injury localisation, detection of concurrent injuries, preoperative injury grading, surgical planning guidance, and comparison with the contralateral uninjured limb for operative planning [18] (Figure 5).

## 7. Crystal Arthritis of the Hip

**Figure 6:**
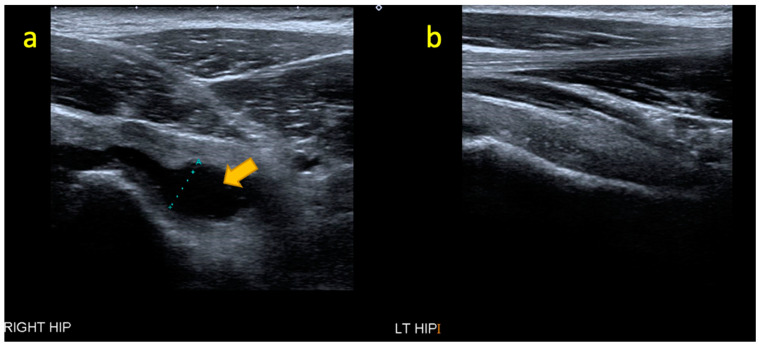
(**a**,**b**) Suspected septic arthritis of the right hip in a 4-year-old boy who subsequently had a surgical washout. Joint aspirate revealed CPPD crystals consistent with acute pseudogout. No pus cells in the aspirate and no organisms were grown. Ultrasound scan image of the right hip in the longitudinal plane shows a large joint effusion, containing a few echogenic debris in keeping with crystal deposition.

Ultrasound in pseudogout shows the pseudo-double-contour sign [19], characterised by calcium pyrophosphate crystal deposits in hyaline cartilage. This differs from gout’s double-contour sign [19,20], where monosodium urate crystals accumulate on articular cartilage surfaces. Point-of-care ultrasound (POCUS) can differentiate pseudogout and gout, therefore reducing the need for joint aspiration and minimising infection risk in patients with known crystalline arthropathies [21] (Figure 6).

## 8. Osteomyelitis Iliac Crest

**Figure 7:**
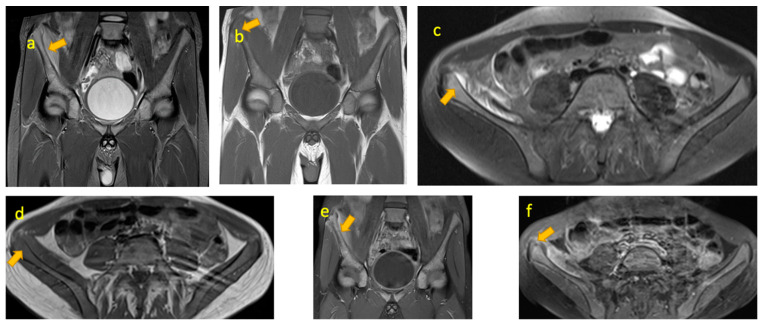
(**a**–**f**) Coronal STIR, coronal T1W, axial T2W FS, and axial T1W MRI images of a 12-year-old boy with acute osteomyelitis of the right iliac crest apophysis. MRI images depict bone marrow oedema signal in the right iliac crest adjacent to the apophysis, a small subperiosteal abscess on the medial side, and severe oedema of the right iliacus muscle.

Pelvic osteomyelitis, affecting 1–11% of haematogenous osteomyelitis cases, typically occurs in the ilium due to its large size and rich blood supply [22]. It is most common in ages 7–14 years with male predominance [22]. Staphylococcus aureus is the most common organism (38–46%); a few other causes determined are Salmonella, Streptococcus pneumoniae, and Gram-negative bacilli [22]. Clinical features include hip/thigh pain, fever (in some cases), a hip held in flexion (due to iliopsoas muscle irritation by abscess formation), and preserved passive hip movement with painful movement of extremities. Laboratory findings are elevated inflammatory markers (ESR, raised WBC count) and positive blood/tissue cultures (50–78%), but a negative culture cannot exclude infection. Initial radiographs may be normal; radiographic features can be visible after two to three weeks [22]. US shows deep soft tissue swelling, and radionuclide bone scans can demonstrate early pyogenic infection. CT can be helpful for the early diagnosis of lytic lesions, sequestrum, or fractures. MRI is the gold standard investigation with 97% sensitivity and 94% specificity for pelvic osteomyelitis demonstrating marrow oedema, hyperaemia of bone, and soft tissue and/or intraosseous abscesses (Figure 7).

## 9. Ewing Sarcoma of Femur

**Figure 8:**
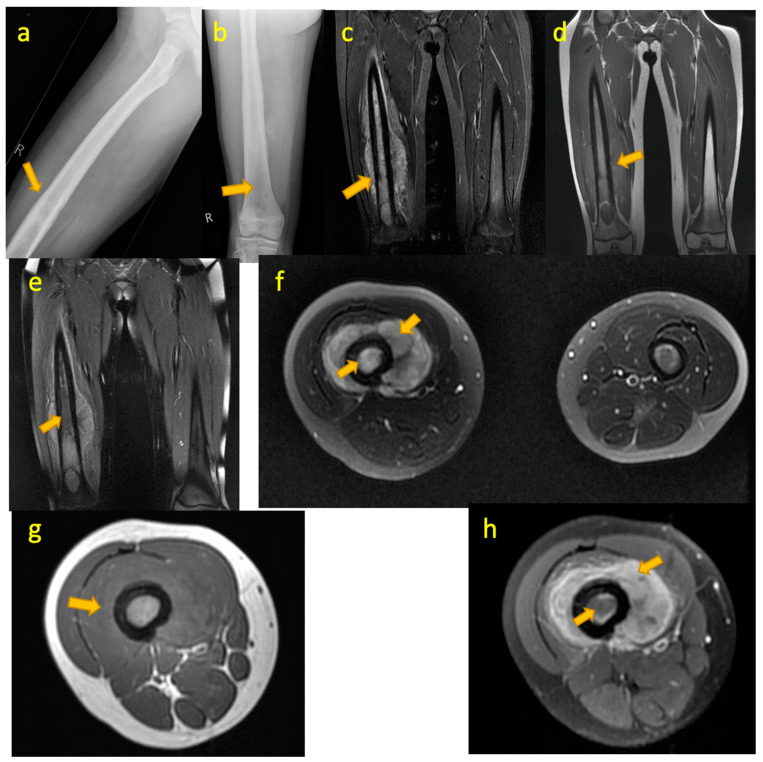
(**a**,**b**) Ewing sarcoma of the right femur in a 13-year-old girl who presented with a painful limp. X-ray of right femur lateral and AP views showing an ill-defined aggressive/permeative bone lesion in the mid and distal femoral shaft with periostitis and soft tissue mineralisation. (**c**–**h**) Coronal and axial STIR, T1W, and post-Gad T1W fat-sat MRI images reveal the extent of the bone lesion and associated large soft tissue component.

Ewing sarcoma is the second most common bone tumour, predominantly affecting adolescents and young adults (median age 15), with peak incidence at 10–15 years of age, predominantly affecting males (M:F 3:1) [23]. Plain radiographs demonstrate destructive moth-eaten lesions, and elevated periosteum showing Codman triangle and onion-skin periosteal reaction. MRI (±CT) with contrast is used for the evaluation of the primary site, extent of the disease, soft tissue oedema, and adjacent organ involvement [24] (Figure 8). Additional imaging modalities used are CT thorax, PET/CT, and MRI (spine and pelvis) for metastasis and metastatic lymph nodes [25]. Open biopsy/CT-guided core needle biopsy (alternative) and molecular cytogenetic analysis of specimen for t(11;22) translocation are needed for establishing a diagnosis [26]. Bone marrow biopsy/marrow aspiration with smear should be considered.

## 10. Langerhans Cell Histiocytosis (LCH) Femur

**Figure 9:**
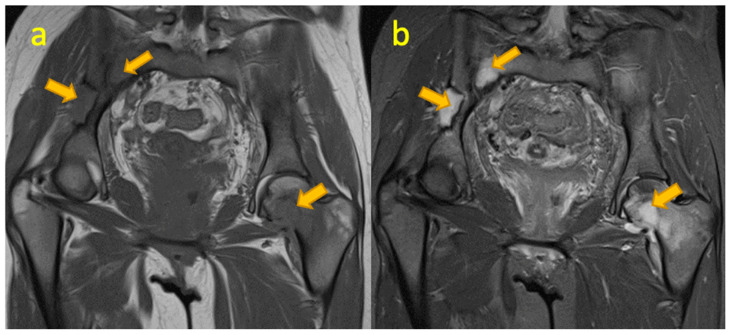
(**a**,**b**) Coronal T1W and STIR MR images of a 16-year-old female with left hip pain. There are multifocal osteolytic lesions in the left femoral neck, right iliac bone, and sacrum, later proved to be Langerhans cell histiocytosis.

A rare disease with multisystem involvement and a heterogenous clinical spectrum. It is caused by uncontrolled monoclonal proliferation of Langerhans cells (distinctive cells of monocyte–macrophage lineage) accompanied by inflammation and granuloma formation. Langerhans cell histiocytosis (LCH) in the pelvis and extremities can mimic infection and malignancy, and therefore LCH should be a consideration in the differential. It frequently presents with aggressive MR features of endosteal scalloping, perilesional oedema, periosteal reaction, and soft tissue mass. The absence of these aggressive features makes LCH unlikely [27] (Figure 9). Differentiation from osteomyelitis is challenging, especially in metaphyseal lesions as the haematogenous spread of infection and abscess can occur at this site [28]. Conversely, a diaphyseal lesion is less likely to be infectious and a soft tissue mass argues against an infective process.

## 11. Leukaemia

**Figure 10:**
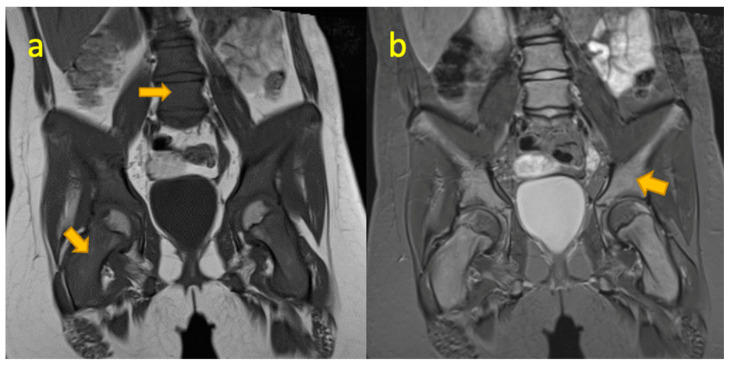
(**a**,**b**) Coronal T1W and STIR MRI images of the pelvis in a nine-year-old girl demonstrate diffuse bone marrow signal abnormality consistent with marrow infiltrative process, later proved to be acute B-cell lymphoblastic leukaemia.

Among all childhood cancers, leukaemia and lymphoma comprise 30% and 8%, respectively [29]. Acute lymphoblastic leukaemia (ALL) comprises 80% of paediatric (2–5 years of age) leukaemia cases (B-cell type 85%, T-cell 15%). Acute myeloblastic leukaemia (AML) accounts for 18% (peaks in second decade). A rare type of leukaemia includes chronic myeloid leukaemia (CML) and juvenile myelomonocytic leukaemia [30]. Given the bone marrow’s role in haematopoiesis, it is a common site for haematologic malignancies. In total, 15–38% of children exhibit musculoskeletal symptoms and 40–75% show at least one radiographic abnormality at presentation. Imaging modalities for evaluating musculoskeletal manifestations of leukaemia and lymphoma include radiography, ultrasound (as a supplement for soft tissue assessment), CT, MRI, 18F-FDG PET/CT, or PET/MRI. Contrast-enhanced MRI is used to confirm viable tumours, areas of necrosis, and the extent of bone marrow infiltration (Figure 10).

## 12. Simple Bone Cyst of Neck of Femur

Unicameral bone cysts (UBCs), also known as simple or solitary bone cysts, are benign, fluid-filled cavities that expand and thin surrounding bone over time.

**Figure 11:**
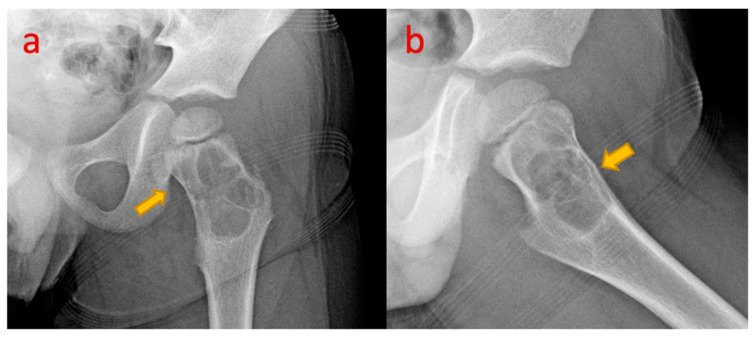
(**a**,**b**) Plain radiograph of the left hip in a 4-year-old boy. AP and frog leg lateral shows a pathological fracture of a bone cyst in the neck of the left femur. Anterior cortical break is appreciated on the lateral projection.

They typically occur in the metaphysis of long bones with open physis and predominantly affect children and adolescents (85%), with a more aggressive and higher recurrence rate in the first decade of life. Common locations include the proximal humerus and femur (90%). These are classified as active if located within 1 cm of the physis and latent if located in the diaphysis [31]. Plain radiography has high diagnostic accuracy and shows a centrally located lesion in the medullary cavity, metaphyseal/juxta metaphyseal, with the long axis parallel to the bone length and geographic appearance with a thin sclerotic margin. Owing to its central location, a cortical break or periosteal reaction (except in fractures), or soft tissue involvement is rare [32] (Figure 11). Diaphyseal simple bone cysts are rarely large, and multicameral with slight expansion. A pathological fracture in the cyst shows the “fallen fragment sign” (the fractured fragment moves with posture changes); similarly, gas foci migrate upwards in a “rising bubble sign”, are pathognomonic of a simple bone cyst, and if seen, other modalities are not required for confirmation of the diagnosis [32]. CT is helpful in areas difficult to assess on radiographs such as the pelvis and spine. Differential diagnoses include aneurysmal bone cyst (ABC), fibrous dysplasia (FD), enchondroma, and intraosseous ganglia.

## 13. Septic Arthritis of the Sacroiliac Joint

**Figure 12:**
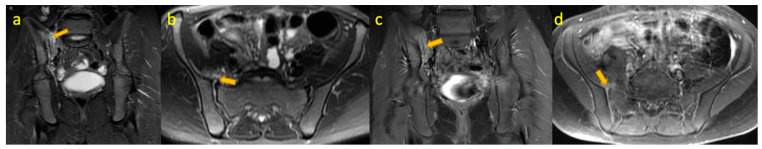
(**a**–**d**) Coronal STIR, axial T2W fat-sat, and post-contrast T1W fat-sat MRI images in coronal and axial planes of a 9-year-old girl showing joint effusion, capsular distension, and enhancing anterior pericapsular soft tissue oedema of the right sacroiliac joint consistent with septic arthritis.

Infectious sacroiliitis is a rare condition in children, affecting only 1.5–4.3% of paediatric osteoarticular infections [33,34]. However, delayed diagnosis can result in long-term morbidity and irreversible joint deformity. Symptoms, including fever, limp, pain, and raised inflammation markers [34], are nonspecific and often overlap with other conditions like appendicitis, hip septic arthritis, and spondylodiscitis. Consequently, imaging plays a crucial role in accurately diagnosing infectious sacroiliitis, guiding clinical decisions, and improving patient outcomes. Magnetic Resonance Imaging (MRI) is the reference standard for assessing the extent and complications of infectious sacroiliitis. Typical MRI features include bone marrow oedema around joints (with/without erosions), effusions in the joint, extracapsular oedema, and abscess in surrounding soft tissues [35] (Figure 12).

## 14. Lumbar Spine Discitis

**Figure 13:**
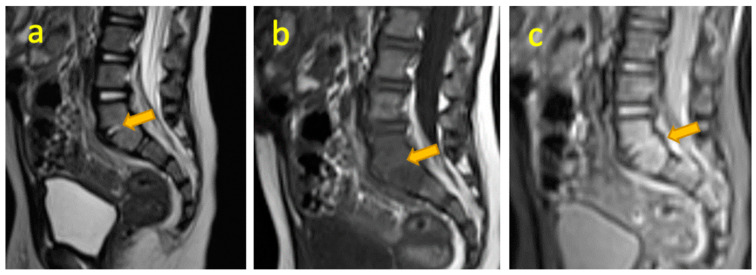
(**a**–**c**) A 13-month-old boy presented with a painful limp for 3 weeks and had raised inflammatory markers on a blood test. Sagittal STIR, T1-weighted, and T1W post-contrast fat-saturated images demonstrate acute L5-S1 intervertebral discitis.

Discitis is an inflammation of intervertebral discs, rare in the young. It affects children of 2–5 years of age. Presenting symptoms include back pain, limping/refusal to walk, irritability, spinal tenderness, and hip stiffness. Most patients have had any of the above symptoms one month before a diagnosis is made [36]. In pyogenic bacterial infections, laboratory tests often show only mildly raised inflammatory markers. Radiographic findings like a narrowed intervertebral disc space and adjacent vertebral endplate erosions may not be evident until 2–3 weeks into the illness. However, spinal radiographs may remain normal even after prolonged disease as described by Scheuerman et al., who studied 52 patients with discitis [37]. Untreated cases may show osteolysis and progress to bony ankylosis. Advanced imaging techniques offer better diagnostic accuracy. FDG-PET distinguishes infections from degenerative spinal changes [38]. MRI is the preferred method for diagnosing discitis (D) and spondylodiscitis (SD), with a sensitivity of 96%, specificity of 93%, and accuracy of 94% [39] (Figure 13).

## 15. TB(Tuberculous) Pyomyositis Thigh

**Figure 14:**
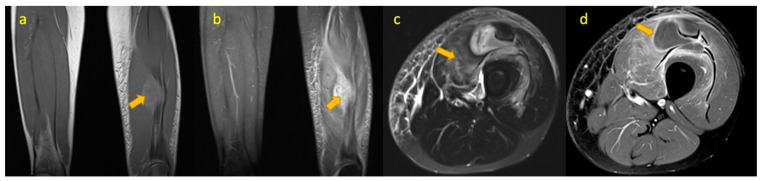
(**a**–**d**)—TB pyomyositis in a 14-year-old boy. Coronal T1W, coronal STIR, axial T2W FS, and post-contrast axial T1W FS images demonstrate TB pyomyositis within the quadriceps muscles of the left mid-thigh. Post-contrast image depicts a non-enhancing intramuscular abscess within the vastus medialis.

In TB, endemic areas of mycobacterial disease should be considered in immunocompetent children with atypical presentation. Mycobacterial pyomyositis can occur through contiguous spread (local transmission), haematogenous spread (bloodborne transmission), direct inoculation, and traumatic inoculation during vaccination. Skeletal muscles are considered “forbidden tissue” for tuberculosis bacteria growth and multiplication [40] due to low oxygen levels, high lactic acid concentration, and paucity of immune cells (reticuloendothelial cells) [41]. Tuberculous pyomyositis, a rare condition, has been sporadically reported in adults and children. Adult cases are associated with miliary tuberculosis, chronic renal failure [42], and renal transplant [42], a manifestation of immune reconstitution syndrome in HIV-AIDS patients [43]. There are few reported paediatric cases in immunocompetent adolescents: a 15-year-old boy with soleus muscle and inguinal lymphadenitis involvement [44] and a 15-year-old Bangladeshi boy with isolated quadratus lumborum muscle infection [45] (Figure 14).

Tuberculosis bacteria lack proteolytic enzymes, leading to an inability to cause pyogenic (pus-forming) infections; however, secondary infections can occur, leading to abscesses in surrounding tissues. MRI distinguishes M. tuberculosis myositis from pyogenic myositis by low T1, high T2 signal intensity in a single muscle with peripheral rim showing subtle hyperintensity on T1, hypointensity on T2WI, and post-gadolinium peripheral rim enhancement in all cases [46]; there is an absence of cellulitis and venous thrombosis surrounding the muscle [47], unlike in the cases of pyogenic myositis.

## 16. Stress Fracture of Sacral Ala

**Figure 15:**
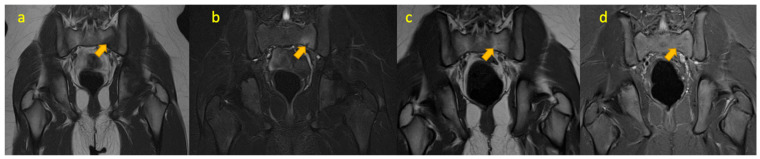
(**a**,**b**) Acute sacral insufficiency fracture due to vitamin D deficiency in a 12-year-old boy. Coronal T1-weighted and STIR MRI images demonstrate a left-sided sacral fracture surrounded by severe bone marrow oedema. image (**c**) coronal T1W image and image (**d**) coronal STIR images show post treatment imaging response with fatty marrow conversion.

Multiple risk factors contributing to stress fractures include vitamin D (25(OH)D) deficiency, female gender, white ethnicity, older age, lower aerobic fitness, prior physical inactivity, excessive training, thin bones, iron deficiency, menstrual disturbances, and inadequate calcium intake [48]. Stress fracture in athletes is not uncommon and most affected athletes are under 25 years of age. The most common site is the lower extremity, which includes the tibia, metatarsals, and pelvis [48]. Rare instances of sacrum involvement are reported [49,50] (Figure 15). Various studies proposed low levels of 25(OH)D and an increased risk of stress or insufficiency fractures [51,52]. Daily supplementation of 800 IU 25(OH)D and 2000 mg calcium can reduce the occurrence of stress fractures. Initial radiographs can be negative for stress fractures. CT, MRI, and Technetium-99 bone scintigraphy help diagnose stress fractures [53].

## 17. Testicular Torsion

**Figure 16:**
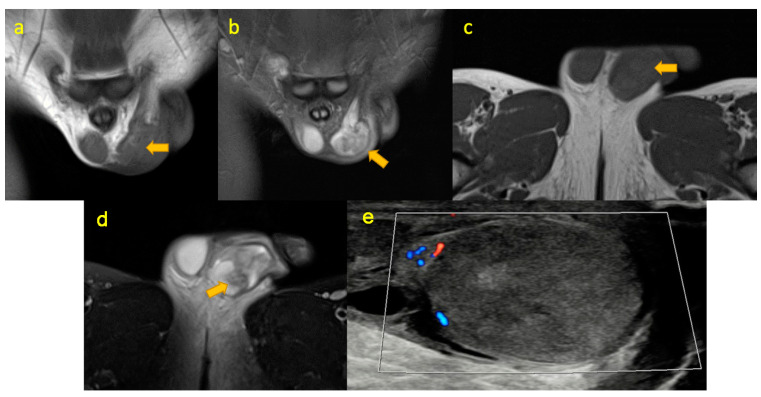
(**a**–**d**) Acute torsion (arrow) of the left-sided testicle in a 13-year-old boy with developmental delay who presented with a painful left-sided limp. Coronal T1W, coronal STIR, axial T1W, and axial T2W FS MRI images show asymmetric enlargement and heterogeneity of the left testicle. Note subcutaneous soft tissue oedema in the left hemiscrotum. (**e**)—colour Doppler ultrasound image in the longitudinal plane shows heterogeneous echogenicity of the left testicle and absence of normal blood flow consistent with acute torsion.

In cases of acute scrotum [54], distinguishing testicular torsion from other treatable conditions (e.g., epididymo-orchitis) is crucial [55,56]. Timely surgical intervention is vital in testicular torsion, as delays can lead to irreversible damage due to impaired blood flow [56]. Initial evaluation by colour/power Doppler US has reported a sensitivity of 89% and a specificity of 100% in the diagnosis of torsion [57], but it is not accurate and conclusive [58]. Atkinson [59] and Luker [60] suggested US colour Doppler exclusively should not be used for torsion in prepubertal cases and MRI with the Dynamic Contrast-Enhanced (DCE) subtraction technique used for detailed testicular perfusion by evaluating contrast enhancement [61] for diagnostic accuracy. Radiograph and US hip were normal in our patient and MRI was performed to look for any signal changes in soft tissue/bone and testicular torsion was then detected; therefore, MRI can be suggested in unidentified causes of limp on other imaging modalities (Figure 16).

## Figures and Tables

**Table 1:** Age-wise common aetiologies of limp in children [1].

Toddler (Less Than 3 Years)	Child (3–10 Years)	Adolescent (11–19 Years)	Any Childhood Age
Cerebral palsy Congenital talipes equinovarus (Clubfoot) Congenital Achilles contracture Enteroviral infection (hand–foot-and-mouth disease) Immunisation reaction Limb-length discrepancy non-accidental trauma Toddler’s fracture Congenital vertical talus	Popliteal fossa cyst (Baker cyst) Köhler disease Dermatomyositis Myositis Legg–Calvé–Perthes disease Leukaemia Coalition of tarsals	Chondromalacia patellae Overuse syndromes Hypermobility syndrome Slipped capital femoral epiphysis (SCFE) Osgood–Schlatter disease Osteochondritis dissecans Sprain (or) strain Tendinopathies Tumour	Fracture Foreign body in foot Transient synovitis Cellulitis Contusion Septic arthritis Developmental dysplasia of hip (DDH) Reactive arthritis Juvenile idiopathic arthritis (JIA) Lyme ‘s disease arthritis osteomyelitis

*Am Fam Physician*. 2023; 107(5): 474–485.

**Table 2:** Table showing imaging features of various pathologies causing limp.

	Juvenile Idiopathic Arthritis (JIA)	Haemophagocytic Lymphohistiocytosis (HLH)	Tuberculosis Pyomyositis	Chronic Recurrent Multifocal Osteomyelitis (CRMO)	Ewing Sarcoma	Langerhans Cell Histiocytosis
**Musculoskeletal imaging features: plain film or CT**	Soft tissue swelling; Osteopenia; Loss of joint space; Erosions; Growth disturbances (epiphyseal overgrowth or “ballooning”); Joint subluxation.	Musculoskeletal radiographic manifestations of HLH are nonspecific and overlap with infectious, inflammatory, and neoplastic disorders. They include the following:	Soft tissue swelling. May be associated with other MSK manifestations of TB; e.g., eccentric lytic bone lesion with minimal periosteal reaction as in TB osteomyelitis.	Lesions can range between purely osteolytic, osteolytic with a sclerotic rim, mixed lytic and sclerotic, and purely sclerotic.	Very variable but usually clearly aggressive. Common findings:	Depends on the phase in which the lesion is imaged (e.g., healing phase) and the affected part of the body. It typically appears aggressive but respects growth plates. CT better demonstrates cortical erosions and soft tissue involvement.
				Early stages: osteolytic lesion; Later stages: progressive sclerosis.	Permeative pattern; Lamellated periosteal reaction (onion skin); Sclerosis.	
**Musculoskeletal imaging features: MRI**	Synovial hypertrophy, joint effusions, ‘Rice bodies’—multiple small loose intra-articular bodies that macroscopically resemble polished grains of white rice, Osseous and cartilaginous erosions, Active synovitis can be characterised by enhancement on T1-weighted gadolinium contrast studies.	Pathological fractures; Diaphyseal periosteal reaction of the long bone; Imaging features of osteonecrosis; Polymyositis (extremely rare).	T1—lesion hypointense; T2—lesion hyperintense; There is almost always abscess formation; Peripheral abscess wall generally subtly hyperintense on T1W images and hypointense on T2W → this is thought to be related to oxygen free radicals and iron within macrophages in the wall of the abscess; After gadolinium contrast injection, peripheral rim enhancement in the abscess wall is often observed; Associated cellulitis and osteoarticular involvement may be present.	Bone marrow oedema; Soft tissue oedema; Extension across the physis; Areas of post-gadolinium enhancement; Periostitis whole-body STIR sequences are useful for determining the extent of disease, including disease sites that may be clinically occult and can be used for subsequent follow-up.	T1—low to intermediate signal; T2—may see hair on end low signal striations; T2—heterogeneously hyperintense signal; T1 + C (gadolinium)—prominent but heterogenous enhancement in adjacent soft tissue.	T1—typically hypointense to isointense; T2—hyperintense; STIR—hyperintense; T1 + C (gadolinium): often shows diffuse contrast enhancement; DWI—demonstrates diffusion restriction.
**Comment**	Hepatosplenomegaly may be seen on abdominal radiographs, and pericardial or pleural effusions may be seen on chest radiographs in ‘Still disease’, i.e., systemic onset JIA.	Other organ systems are affected with their own manifestations, e.g., common chest radiograph findings include alveolar-interstitial opacities with pleural effusions, often with rapid evolution and resolution.	Predilection for the muscles of the pelvic girdle (the spine is generally the most common site of MSK involvement in TB). MRI is the preferred imaging method. Presentation may be with a soft tissue mass that has been present for a long time and constitutional symptoms, with this mimicking a malignant soft tissue mass.	Findings that should raise the possibility of haematogenous osteomyelitis over CRMO include the following: abscess, fistulous tracts, bony sequestrum, and large fluid collection.	Tumour within the long bones is almost always metadiaphyseal or diaphyseal. Ewing sarcomas demonstrate increased uptake on all three phases of Technetium99m methylene diphosphonate bone scans.	Imaging features are not pathognomonic and tissue diagnosis is usually required for a definitive diagnosis. Common lesion locations are the skull and long bones.
